# CT-based deep learning for preoperative prediction of pathological grading of renal clear cell carcinoma

**DOI:** 10.3389/fonc.2025.1656244

**Published:** 2025-10-17

**Authors:** Zhenyu Cui, Tao Ma, Kun Liu, Bingye Shi, Wenzeng Yang

**Affiliations:** ^1^ Department of Urology, Affiliated Hospital of Hebei University, Baoding, Hebei, China; ^2^ Hebei University, Baoding, Hebei, China; ^3^ Department of Ultrasound, Affiliated Hospital of Hebei University, Baoding, Hebei, China

**Keywords:** artificial intelligence, deep learning, clear cell renal cell carcinoma, pathological grading, preoperative prediction

## Abstract

**Objective:**

To construct a noninvasive preoperative prediction model for WHO/ISUP grading of renal clear cell carcinoma (ccRCC) using deep learning combined with four-phase CT images, and to evaluate its efficacy.

**Methods:**

A retrospective study was conducted on 158 ccRCC patients (124 low-grade, 34 high-grade) from the Affiliated Hospital of Hebei University (January 2022-June 2024). Patients were randomly divided into training, validation, and test sets at an 8:1:1 ratio. Four-phase CT images were preprocessed (rectangular box annotation of tumor region of interest [ROI], image resizing to 224×224 pixels). The ResNet34 model was first built to predict ccRCC grading, with performance evaluated by accuracy (ACC) and area under the receiver operating characteristic curve (AUC). The model was then optimized by integrating the SENet attention mechanism (forming the SE-ResNet34 model), and performance before and after optimization was compared.

**Results:**

ResNet34 models based on corticomedullary, parenchymal, and excretory phase images achieved ACC >0.8, with the parenchymal phase model showing the best performance (ACC = 0.867, low-grade AUC = 0.857, high-grade AUC = 0.853). After adding the SENet attention mechanism, the SE-ResNet34 model exhibited improved performance: ACC increased to 0.878, low-grade AUC to 0.929, and high-grade AUC to 0.927.

**Conclusion:**

The SE-ResNet34 model based on parenchymal phase CT images has excellent ability to differentiate WHO/ISUP grades of ccRCC, providing an effective noninvasive auxiliary tool for preoperative pathological grading prediction in clinical practice. However, the model’s robustness and multi-center applicability need further validation before clinical use.”

## Introduction

Renal cell carcinoma (RCC) is the most common renal malignancy with the highest mortality in the urinary system. RCC is a heterogeneous disease divided generally into three major groups according to histopathological grading: clear cells renal cell carcinoma (ccRCC), papillary renal cell carcinoma (pRCC), and chromophobe renal cell carcinoma (chRCC), of which ccRCC is the most common accounting for about 70%-90%. Histopathological grading has been widely demonstrated to be an independent prognostic factor for RCC, with a higher grade indicating higher tumor malignancy and worse prognosis (1, 2). In 2012, the International Society of Urological Pathology (ISUP) reached a consensus at the Vancouver Conference that the prominence of the nucleolus should be emphasized when deciding on grading, and a new grading system (ISUP grading system) was proposed, which is also divided into grades I-IV. Grades I-III were discriminated only by the prominence of the nucleoli, and grade IV was defined by extreme nuclear pleomorphism, multinucleated giant cells, rhabdoid or sarcomatoid differentiation. It is considered that this grading system is only applicable to two histological types of ccRCC and pRCC (3). The ISUP grading system was later recommended by the World Health Organization (WHO) in 2016 with an emphasis on the nucleus staining properties (basophilic nuclei in grade I and eosinophilic nuclei in grades II and III), and was renamed the WHO/ISUP grading system (4). The newly named system provides a better assessment of the prognosis of ccRCC.

With the rapid development of computer hardware and artificial intelligence (AI) theory in recent years, machine learning (ML) and deep learning (DL) have been widely applied in radiological image processing (5). DL is a branch in the development of ML technology that can mimic the human brain in processing complex data through multiple layers of artificial neurons. Specifically, it takes the original image as input and applies multilayer transformation to calculate the output signal, which can automatically develop the optimal model with the best distinguishing features according to the input data for target classification (6). Existing studies have validated the utility of DL in various clinical settings, such as the differentiation of benign and malignant renal tumors (7), differentiation of histological subtypes of RCC (8), and prognosis prediction of RCC (9). However, fewer studies have been conducted regarding the use of DL to predict the pathological grading of ccRCC. In this study, a deep learning model based on CT images was constructed for preoperative non-invasive prediction of histopathological grading of ccRCC, so as to facilitate clinicians to select more effective treatment options and judge the prognosis of ccRCC. The main contributions of this study are as follows: We are the first to combine the SE attention mechanism with ResNet34 to construct a CT parenchymal-phase-based prediction model for clear cell renal cell carcinoma (ccRCC) pathological grading. This integration significantly enhances the predictive performance for high-grade tumor. We systematically compare the performance of models trained using four individual-phase CT images (non-contrast phase, corticomedullary phase, parenchymal phase, and excretory phase) as well as the combined four-phase images. The results confirm that the parenchymal phase serves as the optimal input for the model, which provides practical guidance for optimizing clinical CT scanning protocols in the context of ccRCC diagnosis. Given the constraints of a small dataset, we adopt standardized rectangular region of interest (ROI) labeling and a class-weighted loss function, which collectively enable stable training of the deep learning model. This methodological approach offers a valuable reference for other studies focused on developing medical artificial intelligence (AI) models using limited sample sizes.

## Materials and methods

### General data

Patients who underwent surgical treatment in the Department of Urology of Affiliated Hospital of Hebei University from January 2022 to June 2024 and had a pathologically confirmed diagnosis of clear cell carcinoma in the postoperative kidney bulk specimen were identified through the hospital medical record system and the electronic pathology query system of the Department of Pathology. The name, gender, age, hospitalization number, pathology number, tumor side, body mass index (BMI), hypertension, diabetes mellitus, and maximum tumor diameter of these patients were recorded. Then, the general data of these patients were entered into the picture archiving and communication systems (PACS) of our hospital, and the query was made based on name and hospitalization number to find out whether they had a stage IV renal CT scan examination in our imaging department. This study was approved by the ethics committee of Affiliated Hospital of Hebei University (No.: HDFY-LL-2022-087; date: February 28, 2022), and the data used were analyzed retrospectively, informed consent was waived for all patients.

Inclusion criteria: (1) Patients who had not received treatment such as chemotherapy and surgery before CT scan; (2) Patients who had preoperative CT scan images of four stages: plain phase, corticomedullary phase, renal parenchymal phase and excretory phase, and clear CT images without artifacts; (3) Patients with a histopathological type of clear cell renal carcinoma (ccRCC) and pathological grading according to WHO/ISUP grading criteria; (4) Patients with complete clinical data. Exclusion criteria: (1) Patients with incomplete or poor image quality and artifacts in preoperative four-phase CT images; (2) Patients with a pathological type of non-renal clear cell carcinoma; (3) Patients with tumor metastasis. After a strict screening of inclusion and exclusion criteria, 158 patients were finally enrolled in the study, including 124 patients of grades I-II and 34 patients of grades III-IV.

### CT data

All patients underwent CT four-phase imaging examination using our 64-row CT (GE Discovery CT750 HD) scanner and were instructed to fast for 6-8h before scanning. A non-ionic contrast agent, ioversol, was used at a dose of 1.0-1.2 ml/kg body mass, which was injected via the elbow vein by a double-barrel high-pressure syringe at a flow rate of 3.0-3.5 ml/s. The four CT scans were performed in the following order: plain phase, corticomedullary phase, renal parenchymal phase and excretory phase. The scanning time was before the injection of contrast agent ioversol for the plain phase, 30-35s after the injection of contrast agent for the corticomedullary phase, 50-60s for the renal parenchymal phase, and 180s after the injection of contrast agent for the excretory phase. All four-phase images obtained were exported in the format of digital imaging and communications in medicine (DICOM). Four-phase scanning parameters and conditions: layer thickness 5 mm, pitch 0.984:1, scanning field of view 36 cm×43 cm, matrix 512×512, tube voltage 100–120 kV, tube current 134–409 mA, window width 290 HU, and window level 40 HU.

### Pathological data

All renal tumor specimens were fixed in 10% formalin solution, sent to the pathology laboratory, embedded in paraffin, and cut into 4μm thick sections. After staining with hematoxylin and eosin, the specimens were microscopically diagnosed as renal clear cell carcinoma by two experienced pathologists and were histopathologically graded according to WHO/ISUP grading criteria as shown in **Table 1**, which were classified as grades I-IV. In this study, nuclear grading of I-II was defined as low grade and nuclear grading of III-IV as high grade (10).

**Table 1:** WHO/ISUP grading criteria.

Grading	Definition
Grade I	Absent or inconspicuous nucleoli under 400× microscope, presenting basophilia
Grade II	Obvious nucleolus under 400× microscope, presenting basophilia; visible but not prominent under 100× microscope
Grade III	Obvious nucleolus under 400× microscope, presenting basophilia
Grade IV	Extreme nuclear pleomorphism, multinucleated giant cells, and/or rhabdoid and/or sarcomatoid differentiation

### Image preprocessing

All four-phase CT images exported from our hospital’s PACS system were in DICOM format and opened using the software Raniant DICOM viewer to convert to BMP format. The images in BMP format are losslessly compressed and have good image quality. All CT images were then screened and stored in a folder named after the patient. The screening criteria are as follows: (1) inclusion of all tumor dimensions; (2) clear visibility of the tumor without volume effect.

**Figure 1:**
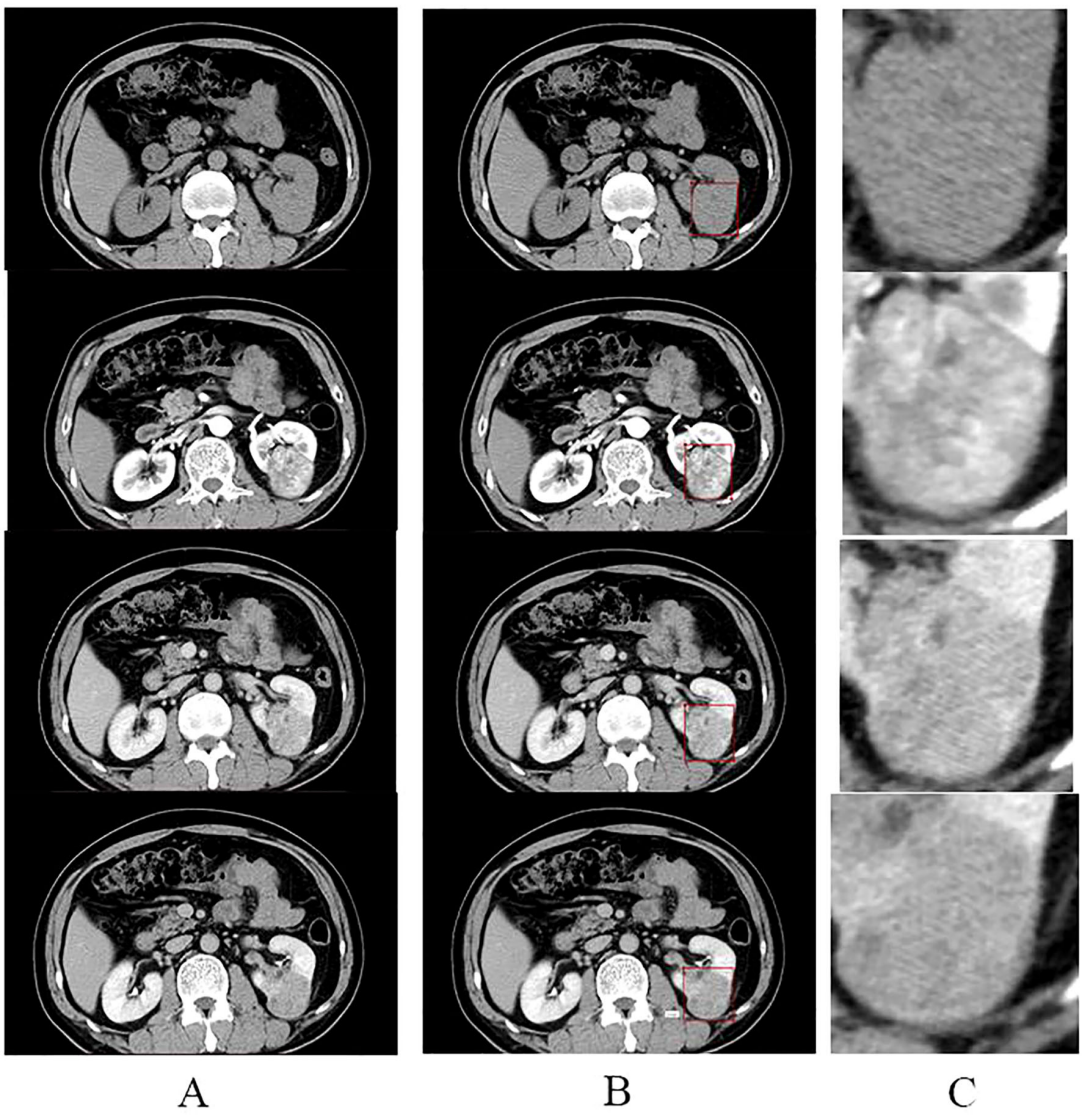
Annotation and cropping of four-phase CT images. **(A)** shows the four-phase CT images of the plain, corticomedullary, parenchymal, and excretory phases, from top to bottom, respectively. **(B)** shows the corresponding four-stage CT images that have been annotated in the rectangular box. **(C)** shows images in the center of **(C)** where the tumor area annotated in the rectangular box of **(B)** was cropped to remove the background and enlarged.

All screened four-stage CT images were loaded into labelm (http://labelme.csail.mit.edu/Release3.0/) and reviewed by two urologists with more than 10 years of experience in urological tumor diagnosis, followed by layer-by-layer rectangular box annotation of the tumor region of interest (ROI), and finally, the generated json file was saved. We opted for rectangular-box ROIs instead of tumor-contour segmentation for reasons of efficiency and clinical practicality. The dataset comprised 5,104 training, 382 validation and 381 test images; using a standardized rule of “expand 1–2 mm beyond the longest diameter”, two senior urologists completed all rectangular annotations within one week with inter-rater reliability ICC > 0.85. By contrast, pixel-level contouring takes 5–10 min per image—more than a ten-fold increase in workload—and is prone to greater inter-observer variability (ICC typically < 0.8). Rectangular boxes can be drawn directly in the clinical PACS without complex algorithms, making the approach easy for clinicians to adopt and facilitating future integration of the model into routine workflow. The criteria for the annotation are as follows: (1) The rectangular box can be delineated by expanding 1-2mm outward with the maximum tumor transverse and longitudinal diameters as the reference. (2) The rectangular box should contain all tumors, and the tumors should be clear; CT images that are unclear should be discarded and not annotated. The images were cropped according to the annotated rectangular box to remove the background information from the non-tumor area to reduce interference(**Figure 1**). The processed images were resized to 224 × 224 pixels. The consistency of the annotation results was verified by the Intraclass Correlation Coefficient (ICC > 0.85), and consensus was reached through negotiation for inconsistent regions.

### Data set construction and data enhancement

The patients recruited were randomly divided into training set, validation set and test set at a ratio of 8:1:1. There were 99 patients of low grade and 27 patients of high grade in the training set, 12 patients of low grade and 4 patients of high grade in the validation set, and 12 patients of low grade and 4 patients of high grade in the test set. Subsequently, the images of patients in each dataset were placed in five opposite folders, corresponding to the plain phase, corticomedullary phase, renal parenchyma phase, excretory phase, and four-phase combined. Each folder has two subfolders under it, corresponding to the low grade and high grade respectively. Ultimately, the number of images corresponding to all folders was: 1260 images in the training set for the plain phase, 1301 images in the corticomedullary phase, 1278 images in the parenchymal phase, 1265 images in the excretory phase, and 5104 images in the four-phase combined; 103 images in the validation set for the plain phase, 92 images in the corticomedullary phase, 92 images in the parenchymal phase, 95 images in the excretory phase, and 382 images in the four-phase combined; 94 images in the test set for the plain phase, 89 images in the corticomedullary phase, 98 images in the parenchymal phase, 100 images in the excretory stage, and 381 images in the four-phase combined. These datasets will be used for the construction of artificial intelligence models.

Several enhancement methods were utilized to augment data from the CT images in the training set, including 30° random rotation, and random flipping, which makes one image into three and triples the data augmentation. Data augmentation is a technique to transform the data and expand the dataset without changing the data label assignment, satisfying the requirement of deep learning models with large sample sizes. Furthermore, each level of CT images is considered as a training sample, and when one of the images is augmented, the image heterogeneity is increased while sharing the same imaging features. To mitigate the impact of class imbalance between low-grade (n=124) and high-grade (n=34) patients, a class-weighted cross-entropy loss function was implemented during model training. This assigned a larger weight to the minority high-grade class, incentivizing the model to improve its learning from the fewer available high-grade samples.

### Selection of network model

A convolutional neural network, as a network commonly used for processing images in deep learning, presents a structure of multiple layers of complex neural network layers. Residual Neural Network (ResNet) is a classical model of a convolutional neural network. By adding residual connections to the structure, ResNet effectively solves the problem of gradient disappearance and network degradation caused by the increase of network depth of the convolutional neural network, thereby improving the network performance. ResNet34 consists of multiple stacked residual units, pooling layers and fully connected layers. The residual units are composed of a convolutional (conv) layer, a batch normalization (BN) layer, a rectified linear unit (Re LU) function, and a jump connection. The convolution layer is used for feature extraction, the pooling layer is mainly used for feature dimensionality reduction and data concentration, while the fully connected layer plays the role of classification.

### Model training

Model training is divided into two stages: training and validation, with the former using the training set data and the latter using the validation set data. The validation set data is used to adjust the hyperparameters of the model in order to obtain the best model. In general, models are trained using traditional methods and ResNet34 networks are pre-trained in ImageNet. The pre-trained network strategy, also known as migration learning, boasts of speeding up the network training and improving the model accuracy. In this study, images of four different individual phases and four-phase combined from the training set were input to a pre-trained ResNet-34 network in order to train the ResNet-34-based plain phase model, corticomedullary phase model, parenchymal phase model, excretory phase model, and four-phase combined model, respectively. All network training was performed using the stochastic gradient descent (SGD) method with a loss function of cross entropy. In each model training, each image input was counted as a loss function, so as to find the gradient confidence parameters. As the number of training sessions increased, the loss function showed a decreasing trend and eventually leveled off. The images from the validation set were then input to each model to further adjust the hyperparameters of the model. The final hyperparameters of the training models in this study are: learning rate = 0.001, batch size = 32, and the number of optimizations = 50.

For all classification models in this study, the experimental environment was under the training environment of Ubuntu 18.04 with an NVIDIA GeForce RTX 2070 GPU, the SE-ResNet34 model takes approximately 45 minutes per epoch and about 37.5 hours for the full 50-epoch training, while a single-image prediction takes only 0.02 seconds. Memory usage is roughly 4.2 GB during training and 1.8 GB during inference. A scalability test indicates that expanding the dataset to 500 cases would increase training time to around 120 hours, which can be reduced to under 30 hours through multi-GPU parallel training.

### Model testing

In the testing process, the test set data were input to the five prediction models that had been constructed to predict the probability of low-grade and high-grade ccRCC images, respectively, and the sum of the two probabilities was 1. The image with the higher probability was used as the output result of the model. The accuracy, sensitivity, and specificity of the model are calculated based on the correctness of the output results to evaluate the performance of the model, which results in the best model constructed by ResNet-34 on the different contemporaneous data sets.

### Model optimization and testing

To assess the possibility of further improving the predictive performance of the network model, the ResNet34 network was optimized by introducing the SE attention mechanism module based on its structure to obtain the SE-ResNet34 network. The attention mechanism module is derived from the characteristics that the human visual system will focus attention on important areas in the scene. Its core point is to assign different weights and computational resources to the convolutional network based on the difference in importance of the feature maps so that the task can be completed in an efficient and fast manner.

The optimized SE-ResNet34 network was also trained as described above, and the test set data was then fed into the SE-ResNet34 model for evaluation, using only images from the best phase for both the training and test sets. The accuracy, sensitivity, and specificity of the model were calculated based on the correctness of the output results, and the predictive performance of the model before and after optimization of the ResNet34 network was compared.

### Statistical analysis

All data in this study were statistically analyzed using SPSS (version 26.0) as follows: Kolmogorov-Smimov was employed to test the normality of the measures, and those conforming to a normal distribution were expressed as mean ± standard deviation; count data conforming to a normal distribution were compared for statistical differences between low and high grades using the independent samples t test. Those that did not conform to a normal distribution were expressed as median and interquartile spacing, and independent samples nonparametric test - Mann-Whitney U rank sum test was used to compare whether there was a statistical difference between low and high grades. Categorical variables were tested using the chi-square test. P<0.05 indicates a statistically significant difference. Moreover, the receiver operating characteristic (ROC) curve was used to assess the efficacy of the model in predicting the low and high grades of ccRCC, and the accuracy (ACC) of the test set classification results and the area under curve (AUC) were calculated.

## Results

One hundred and fifty-eight ccRCC patients were recruited in this study, including 124 in the low-grade group and 34 in the high-grade group. See the table below for a comparison of clinical characteristics between the two groups. The results showed that no statistically significant difference was observed in the clinical characteristics of patients such as gender, age, tumor side, BMI, hypertension, and diabetes distribution between low-grade and high-grade ccRCC (P>0.05). Furthermore, tumor size differed among low and high grades of ccRCC, with renal tumors of high pathological grade having a larger size than those of low pathological grade (**Table 2**).

**Table 2:** Clinical characteristics of 158 patients with low grade and high grade ccRCC.

Characteristics	Low grade (n=124)	High grade (n=34)	P
Gender			0.364
Male	81	25	
Female	43	9	
Age (years old)	57.52 ± 8.95	58.94 ± 10.36	0.431
Tumor side			0.194
Left	61	21	
Right	63	13	
BMI (Kg/m²)	25.84 ± 3.50	25.40 ± 3.63	0.514
Hypertension			0.312
Yes	72	23	
No	52	11	
Diabetes			0.635
Yes	28	9	
No	96	25	
Tumor size (cm)	4.30 ± 2.02	7.09 ± 9.79	0.003

The models constructed by the ResNet34 network in the corticomedullary, parenchymal and excretory phase images presented preferable prediction validation, with a prediction accuracy greater than 0.8, while those constructed in the parenchymal phase data demonstrated optimal performance, with a prediction accuracy of 0.867, 0.857 for AUC for patients of low grade, and 0.853 for AUC for patients of high grade, see **Table 3**. The accuracy and sensitivity of the five models constructed were higher for predicting ccRCC at low grades than at high grades(**Table 2**, **3**).

**Table 3:** Classification performance of the model constructed by ResNet-34 for images of different phases.

Phase	Grade	Accuracy	AUC	Precision	Sensitivity	Specificity	F1
Plain phase	Low	0.777	0.808	0.853	0.865	0.450	0.859
	High		0.776	0.474	0.450	0.865	0.462
Corticomedullary phase	Low	0.809	0.846	0.908	0.843	0.684	0.874
	High		0.843	0.542	0.684	0.843	0.605
Parenchymal phase	Low	0.867	0.857	0.910	0.922	0.67	0.916
	High		0.853	0.700	0.67	0.922	0.683
Excretory phase	Low	0.863	0.808	0.86	0.988	0.316	0.920
	High		0.776	0.857	0.316	0.988	0.462
Four-phase combined	Low	0.791	0.706	0.843	0.923	0.604	0.891
	High		0.677	0.702	0.604	0.923	0.593

**Figure 2:**
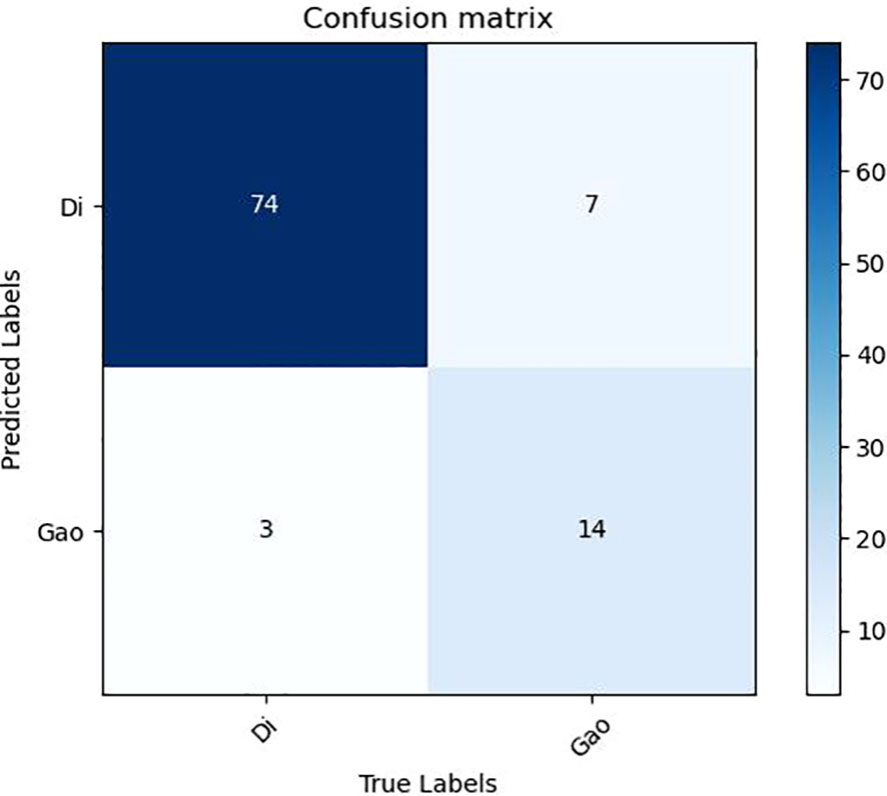
ResNet34 confusion matrix.

**Figure 3:**
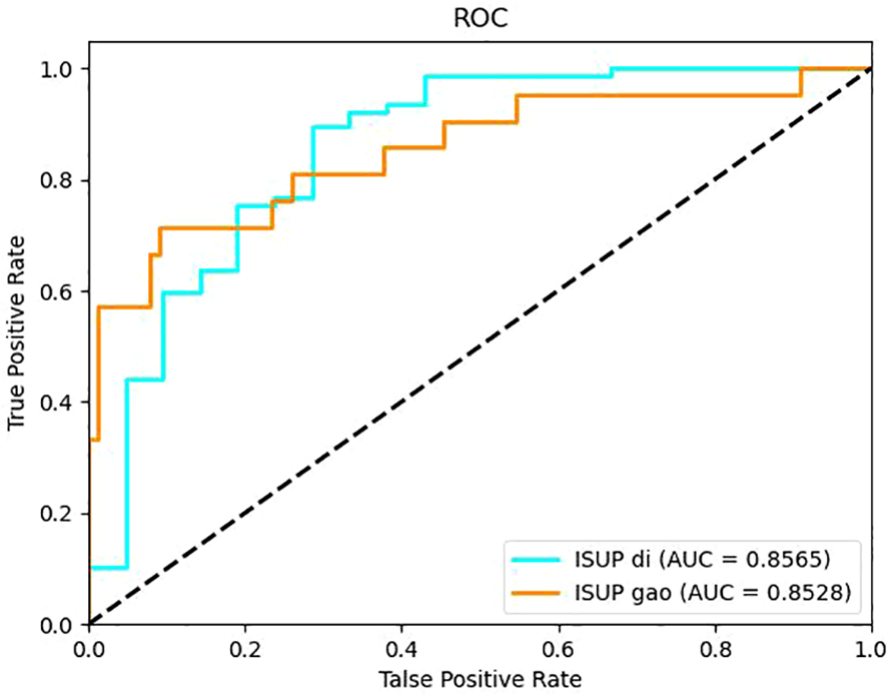
ROC curve of the ResNet34 model built on the parenchymal image set.

**Figure 4:**
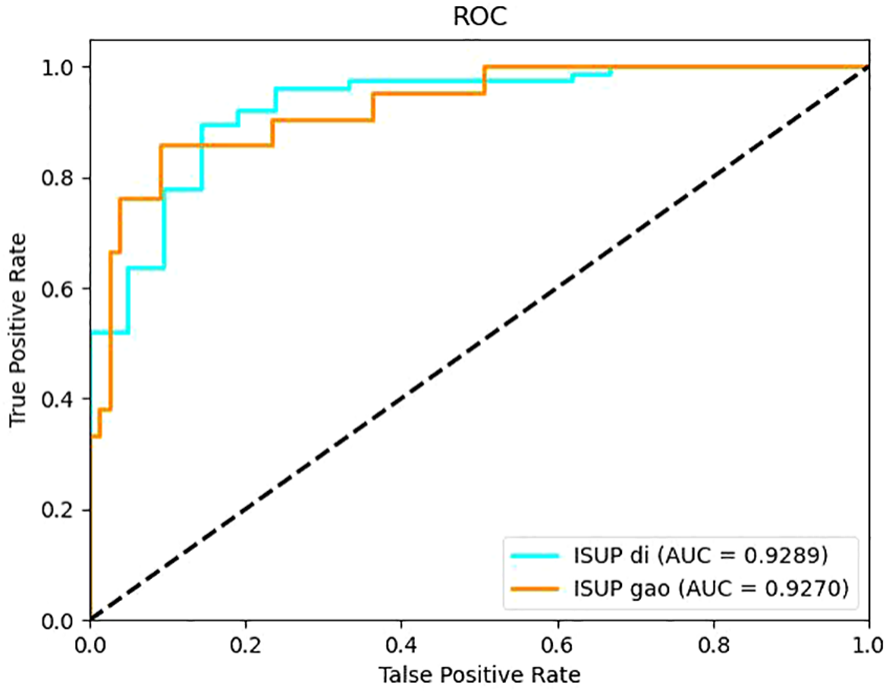
ROC curve of the SE-ResNet34 model built on the parenchymal image set.

It can be seen from the test results of the constructed model that the model constructed by ResNet34 shows the best results in the parenchymal images (**Figure 2**). Therefore, only the parenchymal images in the dataset were used for training and testing the SE-ResNet34 network. The accuracy of the SE-ResNet34 model in the test set was 0.878, which was 0.929 for AUC for patients of low grade, and 0.927 for AUC for patients of high grade, and the ROC curve is shown in **Figures 3**, **4**. After adding the SE attention mechanism, the prediction accuracy of the model improved from 0.867 to 0.878, and that of AUC improved from 0.857 to 0.929 for patients of low grade and from 0.853 to 0.927 for patients of high grade, as shown in **Table 4**, **Figure 4**.The observed higher sensitivity for low-grade predictions may be partially influenced by the inherent class imbalance in the dataset.

**Table 4:** Comparison of accuracy and AUC of ResNet34 model before and after optimization.

Model	Grade	Accuracy	AUC	Precision	Sensitivity	Specificity
ResNet34	Low	0.867	0.857	0.914	0.961	0.667
	High		0.853	0.824	0.667	0.961
SE-ResNet34	Low	0.878	0.929	0.882	0.974	0.524
	High		0.927	0.846	0.524	0.974

## Discussion

Nuclear grading is closely related to the aggressiveness and prognosis of RCC, and if histopathological grading of RCC can be predicted preoperatively, it can guide clinicians to formulate appropriate clinical treatment strategies to a certain extent. Currently, the gold standard for preoperative histopathologic grading of RCC is renal tumor aspiration biopsy. However, this technique is invasive and patients may be at risk of sampling bias and complications, and the accuracy of assessing tissue grading is relatively low. There have been several previous studies on preoperative prediction of ccRCC WHO/ISUP grading using traditional machine learning methods, in which features such as histogram features and texture features are extracted by the region of interest of the tumor outlined in the imaging image for analysis, and the optimal features are extracted using statistical methods, and then a prediction model is built based on the optimal features. Sun X et al. (11) selected the largest cross-section of the tumor to map the region of interest (ROI) based on the CT images of 227 ccRCC patients in both dermal medullary and parenchymal stages, and the support vector machine model constructed by using the optimal features jointly screened in both stages could effectively distinguish the WHO/ISUP grading of renal clear cell carcinoma with an AUC of 0.91. Shu J et al. (12) extracted radiological features from the outlined tumor volume of interest (VOI) and used LASSO to screen the features to obtain the optimal features based on the optimal features obtained from the corticomedullary images, parenchymal images and their combined optimal features, based on 164 low-grade and 107 high-grade ccRCC CT images in both phases. Three machine learning models were constructed based on the optimal features obtained from the dermatoglyphic and parenchymal images and their combined optimal features. It was found that the AUC of the combined corticomedullary + parenchymal model was 0.822, which was higher than that of the corticomedullary and parenchymal models alone. The above studies all used traditional machine learning methods to construct prediction models, indicating that the prediction of ccRCC WHO/ISUP classification based on traditional machine learning methods is a feasible and noninvasive method, and the performance of the machine learning models constructed using multi-phase CT images may be better than that of a single phase. In contrast, traditional machine learning systems require careful engineering and considerable domain expertise to extract features of the input data and thus use statistical methods to combine or separate the data based on these features, which will inevitably lead to a more subjective result with the disadvantages of being time-consuming, labor-intensive, and not fully demonstrating the features of the input data. But deep learning methods can avoid these shortcomings. Deep learning enables automatic extraction of pixel-level image features without the need to design and select features by hand, and a classification system based on deep learning can classify data better compared to manually extracted features, which is the core advantage of the model constructed in this study.

Deep learning-based convolutional neural networks use multiple neural network layers to gradually extract higher-level features from the original input data, reflecting the hierarchical structure of the data, and can be enriched with features by the number of stacked layers (depth). However, as the depth of the neural network model increases to a certain level, the problem of gradient disappearance or gradient explosion can occur, and the model accuracy decreases dramatically without warning, a phenomenon called Degradation, which is not caused by overfitting but by the fact that adding more layers to a model of appropriate depth leads to higher training error. The core of ResNet is the residual units stacked in the network, and each residual unit consists of 2 convolutional layers and a shortcut connection, and the shortcut connection can make the deep network easier to optimize and solve the degradation problem brought by the deep network. ResNet has been widely used in the processing of medical images due to its good network performance, and it has achieved good results in diseases such as lung tumors (13), new coronary pneumonia (14) and breast cancer (15). better results have been achieved in auxiliary diagnosis. There have been attempts to construct ResNet models for predicting the pathological grading of ccRCC based on imaging images. Zhao Y et al. (16) developed a low-level deep learning prediction model for stage I and II ccRCC based on conventional MRI images, which was based on the ResNet50 net structure and achieved an accuracy of 0.88 in the Fuhrman test set and an accuracy of 0.5 in the WHO/ISUP testers with an accuracy of 0.83. Lin F et al. (17) developed a CT-based ResNet model for predicting WHO/ISUP classification of renal clear cell carcinoma using a deep learning approach and found that the use of migratory learning, no attention level settings, and images containing less background information helped to improve the accuracy of the model, with the best model having an accuracy of 77.9% and The AUC was 0.81, but the study chose the largest cross-section of the tumor to map the region of interest (ROI) and did not consider the volume of the tumor. Resnet convolutional neural network mainly has several different complex structures of Resnet18, Resnet34, Resnet50, Resnet101, Resnet150, and the number represents the depth of the network. The larger the number indicates a deeper and more complex network depth, this study was selected to build a model with a relatively simple structure Resnet34 in the Resnet convolutional neural network, and the prediction model constructed achieved good prediction results. Lin F et al. (17). concluded that the model constructed with a simpler network of Resnet layers has better performance in predicting ccRCC WHO/ISUP classification, and the possible The reason is that more complex models require a larger amount of data and relatively small training samples cannot take full advantage of the complex models. In addition, this study was based on layer-by-layer outlining on CT images into 3D VOI, which was different from the 2D ROI of a single sheet selected in some previous studies. 3D outlining can reflect more realistic tumor volume, is more sensitive to the internal heterogeneity of the tumor, and can present the internal characteristics of the tumor more comprehensively, which may be the reason why the accuracy and AUC of the model constructed in this study were higher than those of the model constructed by Lin F et al. (17).

This study also compared the performance of each model for ccRCC disparate CT images. Previously, some researchers used conventional machine learning algorithms to construct prediction models using only plain-phase or single-phase enhanced CT images (18, 19), and although they achieved certain results, the constructed models could not fully utilize the features of the disparate images. Many other researchers have used two- or three-phase CT images to construct machine learning prediction models, and they agree that the performance of the models created by combining multi-phase CT images is better than that of the models created by single-phase images (20). In this study, Resnet34 models were constructed using the scanned, dermatomedullary, parenchymal, excretory, and four-phase combinations, and it was found that the model constructed using the parenchymal CT images had the highest accuracy and AUC, and the performance was even better than that of the model constructed using the four-phase combination, and the worst performance was that of the model constructed in the scanned phase, which was inconsistent with most of the previous results, probably because the previous studies used They manually extracted the optimal features from the CT images of each phase and then fused these optimal features together to build the model, and the performance of the constructed model was largely better than that of the model constructed based on the optimal features extracted from the images of a single phase only. In contrast, this study uses a deep learning algorithm to build the model. Although the deep learning-based Resnet has good performance of automatically extracting image features, it is impossible to avoid automatically extracting the image dominant features while also extracting the image inferior features, and when Resnet extracts the four-phase joint image features, it also extracts some of the inferior features such as the flat-scan phase, thus lowering the overall diagnostic performance of the model. the overall diagnostic performance. The results of this study may be useful for scholars who want to conduct similar studies in the future. In addition, since the model constructed in this study predicts the pathological grading of ccRCC, and the level of pathological grading represents the strength of the aggressiveness of the tumor, clinicians can pay more attention to the CT images of the parenchymal stage when judging the malignancy of ccRCC.

After testing the performance of the Resnet34 model on different CT images, it was found that the performance of the Resnet34 model constructed based on parenchymal stage images was optimal. To explore whether the performance of the model could be further improved, the Resnet34 structure was optimized on the basis of the attention mechanism module SE-Net (Squeeze and Excitation), and the SE- Resnet34 model was formed. The SE- Resnet34 model was then retrained and tested on the CT images of the substantive period, and it was found that the SE- Resnet34 model had an accuracy of 0.878, a low-level AUC of 0.929, and a high-level AUC of 0.927 in the test set, which was a certain improvement in performance compared with that before the improvement. This study is a single-center retrospective study and there may be bias in data selection, so the generalization performance of the model needs to be further validated.

This study has several limitations. Firstly, its retrospective, single-center design and relatively limited sample size (especially for high-grade [WHO/ISUP III-IV] ccRCC cases) collectively constrain the research. On the one hand, the retrospective and single-center nature introduces potential selection bias. The model was developed and validated based on data from a single institution with specific CT protocols, meaning its performance may not be directly transferable to other centers, which severely limits the generalizability of our findings. On the other hand, despite using data augmentation and class-weighted loss functions to mitigate class imbalance, the small absolute number of high-grade tumors remains unresolved. This not only contributes to the observed performance difference between low-grade and high-grade cases but also further impacts the model’s generalizability. Secondly, the lack of an external validation cohort is a significant limitation. Given the constraints of single-center data and limited sample size, the reliability of the model’s performance cannot be fully confirmed. Therefore, future multi-institutional, prospective external validation is essential to verify the robustness and clinical utility of the proposed model, and our findings need to be validated in such a larger cohort. In future work, we will replace the rectangular ROI with a “semi-automatic tumor-contour segmentation” pipeline that comprises three steps:(1) A U-Net model pretrained on our in-house cohort of 158 patients will first generate an initial tumor outline.(2) One senior radiologist will manually refine the auto-segmentation (adjusting only deviating borders); this keeps non-tissue inclusion below 5% while limiting annotation time to 1–2 min per image.(3) We will retrain the SE-ResNet34 classifier with the new precise ROIs and compare its performance with the rectangle-ROI version; we expect the sensitivity for high-grade ccRCC to rise from 0.67 to ≥ 0.80.

The SE-Resnet34 model based on parenchymal CT boasts a preferable differentiation of WHO/ISUP grade of clear cell renal carcinoma, providing an effective auxiliary means for noninvasive preoperative prediction of pathological grading of renal clear cell carcinoma in clinical practice. Future multi-center validation (≥500 cases, GE/Siemens/Philips CT) and prospective trials are required before clinical discussion.

## Data Availability

The datasets presented in this article are not readily available due to the ethical restrictions and privacy protection requirements imposed by the research ethics committee. Requests to access the datasets should be directed to the corresponding author.
